# Suppression of neuropathic pain hypersensitivity by TRPM8 is mediated by mGluR group II and III receptors acting differentially on distinct nociceptor inputs to spinal cord

**DOI:** 10.3389/fphar.2026.1806325

**Published:** 2026-04-14

**Authors:** Rory Mitchell, Liting Sun, Marta Czapranska, Sue Fleetwood-Walker

**Affiliations:** Institute for Neuroscience and Cardiovascular Research, College of Medicine & Veterinary Medicine, University of Edinburgh, Edinburgh, United Kingdom

**Keywords:** analgesia, metabotropic glutamate receptor, mGlu2, mGlu8, neuropathic pain, parallel nociceptive processing, TRPM8

## Abstract

**Background:**

Chronic pain following nerve injury (neuropathic pain), is notoriously difficult to treat, with current analgesics showing limited efficacy and adverse or dangerous side effects. One new candidate analgesic target is the TRPM8 ion channel, identified as the peripheral detector for innocuous cool sensation and reported to attenuate spinal cord pain processing by processes involving inhibitory metabotropic glutamate (mGlu) receptors.

**Methods:**

Highly selective Group II/III mGluR antagonists and allosteric modulators were used in a nerve injury model to identify the specific receptor subtypes mediating TRPM8-mediated attenuation of nociceptive (pain) processing through peptidergic and non-peptidergic afferent inputs (associated with thermal and mechanical nociception respectively). Integrated experimental approaches involved immunofluorescence histochemistry, functional Ca^2+^ fluorescence responses of *ex vivo* synaptoneurosomes and *in vivo* reflex pain behaviours.

**Results:**

Differential expression of TRPV1 and MrgprD was demonstrated in peptidergic and non-peptidergic afferents and their selective activation by capsaicin and β-alanine characterised to interrogate transmission at the first central synapses from these afferents in dorsal spinal cord synaptoneurosomes. TRPM8-evoked attenuation of nerve injury-induced increments in capsaicin responses was selectively modified by mGlu_8_-targetting agents whereas the equivalent effect on β-alanine responses was selectively modified by mGlu_2_-targetting agents. Other Group II/III mGluR subtypes appeared not to be involved. Reflex pain behaviour assessments correspondingly pointed to mGlu_8_ and mGlu_2_ being selectively involved in TRPM8 attenuation of thermal and mechanical hypersensitivity respectively. Spinal administration of mGlu_2_ and mGlu_8_ antagonists impacted TRPM8 attenuation of nerve injury-induced synaptic hypersensitivity at spinal and also supraspinal regions of the CNS associated with pain processing.

**Conclusion:**

Our findings clarify the roles of specific Group II/III mGluRs in the antinociceptive effects of TRPM8 activation against nerve injury-induced hypersensitivity. The mGluRs involved in impacting peptidergic (thermal-associated) nociceptive inputs and non-peptidergic (mechanical-associated) nociceptive inputs appear quite distinct - mGlu_8_ and mGlu_2_, respectively. This provides robust evidence to support the fundamental concept of distinct parallel processing and differential modulation of these classes of inputs. This work extends our understanding of the basis for TRPM8 analgesia, identifies distinct modality-specific processes and points to the possibility of refined therapeutic interventions using mGluR modulators as adjunct promoters of particular elements of analgesia.

## 1 Introduction

There is a major unmet need for safe and effective non-opioid analgesics to treat chronic hypersensitive pain states, especially where nerve damage (leading to altered central processing) is involved. Cloning of the TRPM8 ion channel (McKemy et al., 2002; Peier et al., 2002) revealed its responsiveness to mild cooling and menthol, implicating it as the likely mediator of the long-known antinociceptive effects of peripheral cooling and mint extracts containing menthol and highlighting TRPM8 in peripheral sensory neurons as a potential new target for analgesia.

In chronic pain patients, or pain model volunteers, various reports have described analgesic outcomes following TRPM8 agonist treatment, notably in patients with refractory neuropathic pain (Borhani Haghighi et al., 2010; Brid et al., 2015; Colvin et al., 2008; Cortellini et al., 2017; Davies et al., 2002; Johar et al., 2012; Keshavarzian and Shahgholian, 2017; St Cyr et al., 2015; Stefanelli et al., 2019; Storey et al., 2010; Taylor et al., 2012; Topp et al., 2013). Similar results were found in more extensive exploratory clinical trials (Airaksinen et al., 2003; Fallon et al., 2015; Sundstrup et al., 2014; Zhang et al., 2008). Several randomised clinical trials (NCT01855607, NCT02984072 and NCT04276727) are underway to further evaluate the analgesic efficacy of the TRPM8 agonist, menthol, in neuropathic and other pain states (Kokki and Kokki, 2022; Li et al., 2022).

Initial laboratory studies to address the basis for potential analgesic effects of TRPM8 agonists revealed robust efficacy against both thermal and mechanical hypersensitivity in rat models of both neuropathic and inflammatory pain that was abrogated by TRPM8 antisense knockdown (Proudfoot et al., 2006). These findings were confirmed in the same neuropathic pain model (Su et al., 2011) and corroborated in a number of other pain models in mice, where specific mediation by TRPM8 was demonstrated by gene deletion or a selective antagonist (Liu et al., 2013). Further, the cooling-evoked attenuation of nocifensive behaviours in response to intraplantar formalin or mechanical hypersensitivity in a neuropathic pain model was abrogated by TRPM8 knockout or toxin-evoked ablation of TRPM8-containing neurons (Dhaka et al., 2007; Knowlton et al., 2013). Other studies with TRPM8 agonists administered at various doses and routes also reported an analgesic effect (Galeotti et al., 2002; Gaudioso et al., 2012; Hilfiger et al., 2021; Klein et al., 2010; Pabbidi and Premkumar, 2017; Pan et al., 2012; Wright et al., 2018), although varied interpretations were offered. In some other cases, analgesia was generally not observed (Caspani et al., 2009; Colburn et al., 2007; Ji et al., 2007; Patel et al., 2014). The use of different models and high doses of the TRPM8 agonists may however have contributed to differing results.

Further studies from our laboratory showed that the analgesic effects of TRPM8 agonists could be amplified by the concurrent peripheral administration of 5-HT_1B_ receptor agonists and could be reversed by intrathecal blockers of Group II/III mGlu, but not opioid receptors (Proudfoot et al., 2006; Vinuela-Fernandez et al., 2014). The current study aims to further elucidate the roles of specific individual Group mGluII/III receptors in regulating thermal and mechanical hypersensitivity in a rat neuropathic pain model and thereby provide new insights into potential therapeutic opportunities.

Sensory primary afferent neurons are virtually all considered to store and release glutamate as transmitter (Alvarez et al., 2004; Brumovsky et al., 2007; Li et al., 2003; Usoskin et al., 2015). The nociceptive afferents fall into two main categories; peptidergic C-fibres, which are essential for responses to noxious thermal stimuli and non-peptidergic C-fibres, which are thought to respond preferentially to noxious mechanical stimuli (Basbaum et al., 2009; Joseph and Levine, 2010). A number of channels/receptors are selectively expressed in these. Peptidergic C-afferents selectively express the TRP vanilloid receptor 1, TRPV1, whereas non-peptidergic C-afferents selectively express the Mas-related G protein-coupled receptor D (MrgprD) and can be identified by binding isolectin IB4 (Cavanaugh et al., 2009; Dong et al., 2001; Rau et al., 2009; Reynders et al., 2015; Snider and McMahon, 1998; Zylka et al., 2005). Chemical or genetic ablation of unmyelinated afferents expressing TRPV1 results in marked, selective deficits in noxious thermal detection (Cavanaugh et al., 2009; Karai et al., 2004; Pogorzala et al., 2013). In contrast, ablation of MrgprD^+^ afferents selectively impairs noxious mechanical sensation (Cavanaugh et al., 2009), while some additional deficits in thermal sensing have been reported in MrgprD^−/−^ mice (Rau et al., 2009). Independent optogenetic activation of TRPV1^+^ and MrgprD^+^ afferents elicits aversive behaviours, especially following nerve injury (Beaudry et al., 2017; Warwick et al., 2021). Selective pharmacological agents are available to independently activate each of these classes of nociceptor; capsaicin for TRPV1 (Caterina et al., 1997) and β-alanine for MrgprD, (Crozier et al., 2007; Rau et al., 2009; Shinohara et al., 2004), providing tools to interrogate modulatory events at the first synaptic connections of these afferents by measuring Ca^2+^ fluorescence in dorsal spinal cord (DSC) synaptoneurosomes. Most studies find that TRPM8-positive afferents form a minor group of afferents, of which the vast majority appear quite distinct from those showing classical markers of nociceptors, such as TRPV1, substance P/CGRP or the binding of IB4 lectin (Dhaka et al., 2008; Jankowski et al., 2017; Knowlton et al., 2013; Mitchell and Fleetwood-Walker, 2015; Peier et al., 2002; Usoskin et al., 2015), so appear to run in parallel to nociceptors into the spinal dorsal horn, where this mGluR-mediated inhibitory gating is mediated. How activation of TRPM8 afferents impacts on nociceptive processing through peptidergic and non-peptidergic afferents could therefore potentially be quite distinct.

## 2 Materials and methods

### 2.1 Animals

Animal breeding, maintenance and experimental procedures complied with ARRIVE guidelines and were carried out in accordance with the UK Animals (Scientific Procedures) Act 1986, with approval from the University of Edinburgh’s Local Ethical Review Board. Animals were housed under a 12 h light-dark cycle and given access to food and water *ad libitum*. Experiments were carried out on adult male Sprague-Dawley rats (200–350 g in weight). The sciatic nerve chronic constriction injury (CCI) preparation was used as a model of chronic neuropathic pain in rats (Bennett and Xie, 1988). Animals were anaesthetised with a 4% isoflurane/oxygen mixture (Zeneca, Cheshire, UK) before exposure of the sciatic nerve, proximal to the trifurcation at mid-thigh level. Four loose ligatures of chromic catgut (SMI AG, Hunningen, Belgium) were tied around the nerve, separated by 1 mm. We have used this model regularly for many years (Garry et al., 2005; Garry et al., 2003; Mitchell et al., 2020; Proudfoot et al., 2006) and find that these animals consistently develop ipsilateral hypersensitivity in thermal and mechanical paw withdrawal reflexes, which peaks between days 8 and 12 post-surgery, when the current experiments were carried out. Nerve-injured and control animals were housed in separate groups until experimentation and no further cage randomisation was carried out as the actual experiments were of an acute non-recovery nature.

### 2.2 Quantitative sensory testing *in vivo*

Standard reflex behavioural tests of paw withdrawal to threshold thermal and mechanical stimuli were used (Mitchell et al., 2020; Proudfoot et al., 2006). Thermal nociceptive sensitivity was measured as Paw Withdrawal Latency (PWL; in sec) using Hargreaves’ infrared apparatus (Linton Instrumentation) set to a maximum temperature of 52 °C and a cut-off time of 20 s. Mechanical nociceptive sensitivity was assessed as Paw Withdrawal Threshold (PWT; in g) from force-calibrated von Frey nylon filaments (Stoelting, Illinois) applied in ascending order until explicit brisk paw withdrawal was observed. Testing sessions were always separated by at least 5 min to avoid sensitisation of responses. CCI animals were routinely screened with von Frey filaments to ensure that ipsilateral PWT scores were reduced to at least 4-fold lower than contralateral values before animals were randomly allocated to experimental treatment groups. We routinely find only minor variation in PWT and PWL scores within CCI and control groups and ipsilateral:contralateral normalisation was carried out here to further minimise variability. The behavioural investigator was blinded to drug treatment as the identity of drug solutions was encrypted and known only to the assistant investigator.

### 2.3 Drug administration *in vivo*

In order to produce localised topical activation of TRPM8-expressing afferents, animals were maintained under anaesthesia (4% isoflurane in oxygen) for 15 min, during which the hindpaw ipsilateral to nerve injury was immersed in a solution of the selective TRPM8 activator, icilin (30 μM, submaximal; 200 μM, maximal; in propylene glycol with 0.1% dimethylsulphoxide). The vehicle had no effect alone and control experiments consistently showed no discernible effect of ipsilaterally or contralaterally administered icilin on contralateral paw withdrawal responses. In animals destined for behavioural testing, a recovery period of 15 min was allowed before assessment. When mGluR agents were investigated in such experiments, they were injected intraperitoneally immediately before the start of icilin administration in a volume of 0.2 mL sterile saline with 0.1% dimethylsulphoxide, which had no effect alone. In some experiments, where synaptoneurosome responsiveness was to be assessed, mGluR antagonists or positive allosteric modulators (PAMs) were applied directly to the dorsal surface of the spinal cord at segments L4-L6, following laminectomy and dura removal, under maintained anaesthesia. Drugs were added immediately before the start of topical icilin administration and replenished as necessary for 15 min throughout the full duration of icilin administration. The vehicle was sterile saline with 0.1% dimethylsulphoxide, which had no effect alone.

### 2.4 Immunofluorescence histochemistry

Dorsal root ganglia (DRG) from spinal segments L4-6 were dissected, rapidly embedded in cryo-sectioning medium (Thermo Scientific) and frozen on dry ice. 18 μm sections were cut by cryostat and alternate sections were mounted onto poly-L-lysine coated slides (Thermo Scientific). Sections were washed in Tris-Buffered Saline (TBS) pH 7.60 and then incubated for 1 h at room temperature in blocking buffer (10% normal donkey serum, 4% fish skin gelatin, 0.2% Triton X-100 in TBS) prior to overnight incubation at 4 °C with a combination of primary antibodies in buffer (4% normal donkey serum, 4% fish skin gelatin and 0.2% Triton X-100 in TBS). Primary antibodies used were guinea pig polyclonal anti-TRPV1 (Abcam, ab10295; 1:3000), rabbit polyclonal anti-CGRP (Peninsula T-4239, 1:1000) and rabbit polyclonal anti-MrgprD (Alomone, AMR-061, 1:400). Sections were washed in TBS and incubated for 1 h at room temperature with a combination of extensively cross-adsorbed, fluorescent secondary antibodies or Alexa Fluor-488 conjugated isolectin IB4 (Invitrogen; I21411; 1:3000) in buffer (4% normal donkey serum, 4% fish skin gelatin in TBS). Donkey anti-rabbit or guinea pig secondary antibodies, labelled with 647 or 568 nm-emitting fluorophores and highly cross-adsorbed against other relevant species, were obtained from Biotium via Sigma-Aldrich and were used at a dilution of 1:600. Sections were washed three further times in TBS and then mounted in ProLong® Gold Antifade (Life Technologies). Standard primary antibody omission or blocking peptide controls, wherever possible, confirmed that non-specific staining was minimal. Concentration-dependence of staining and selective association with small diameter cellular profiles was seen in each case.

### 2.5 Confocal microscopy and image analysis

Fluorescence signals were acquired using a Nikon A1R confocal microscope at 1024 × 1024 pixel size frame, 12 bits per pixel images, with a pinhole size 1AU calculated for 488, using objectives ×10 Plan Fluor/NA0.3, or 20X Plan Apo VC/NA 0.8. Emissions for each fluorophore were obtained sequentially to avoid channel bleed-through. Z-stacks were acquired covering the whole thickness of each tissue section (5 μm and 2 μm step size between planes) and maximum intensity projections were generated using ImageJ/Fiji. Whole cells positive for markers of interest were manually counted (using ImageJ/Fiji software plugin) by an investigator who was blind to which fluorophore colour represented which target. Mean percentage expression values were generated over several sections from each of at least 4 separate animals.

### 2.6 Preparation of synaptoneurosomes and measurement of Ca^2+^ fluorescence responses

Synaptoneurosomes, representing re-sealed pre-synaptic and closely apposed postsynaptic elements freshly prepared from CNS tissue (Hollingsworth et al., 1985; Villasana et al., 2006), can be used to evaluate mechanism of synaptic transmitter release and its modulation, ion fluxes and signalling events (Benavides et al., 1988; Dominguez et al., 2007; Ebstein and Daly, 1982; Kamisaki et al., 1993; Schwartz et al., 1985; Shinomura et al., 1999). Using protocols we developed to optimise their metabolic and functional integrity and more precisely evaluate receptor-mediated modulation of synaptic function (Mitchell et al., 2019; Sun et al., 2013; Vinuela-Fernandez et al., 2014) synaptoneurosomes were prepared from L4-L6 DSC or in some cases other CNS regions. Tissue was rapidly removed after cull and immediately homogenised in ice-cold highly oxygenated medium using a hand-held glass homogeniser. The medium was divalent ion-free Krebs-Henseleit buffer, additionally containing HEPES (10 mM, pH 7.4), sodium pyruvate (0.5 mM), glutathione (5 μM), creatine phosphate (2 mM), magnesium chloride (5 mM), sodium kynurenate (300 μM) and Type III protease inhibitor cocktail (Calbiochem, 1:1000). The homogenate was rapidly syringe-filtered through a 30 μm nylon mesh filter (Millipore) and then through a 5 μm pore mixed cellulose fibre matrix filter (Millipore) before centrifugation at 1,500 g for 15 min at 4 °C. The pellet was gently resuspended in ice-cold highly oxygenated medium as above but lacking magnesium ions and kynurenate. Aliquots of synaptoneurosome suspension (150 μL) were dispensed into 48-well plates and Calcium-5 (a sensitive, no-wash Ca^2+^ fluorophore, formulated with an extracellular fluorescence quenching agent; Molecular Devices), was added in 50 μL resuspension buffer containing 8 mM CaCl_2_, before incubation for 45 min at 37 °C under 95% O_2_/5% CO_2_. Plates were transfered to a plate reader (Varioskan Flash, ThermoScientific; thermostatted at 28 °C). Potential modulatory pharmacological agents were then added before synaptic stimuli, such as capsaicin, β-alanine, 5-iodowillardiine or AMPA, as appropriate. Intracellular Ca^2+^ fluorescence was read immediately at excitation 488 nm, emission 518 nm, over 12 × 30 s intervals or 6 × 1 min intervals, taking the mean value from 3 replicate wells for each experimental sample. Each plate included wells with ionomycin (10 μM) and buffer alone controls in order to calibrate the dynamic range of the assay. The investigator for these Ca^2+^ fluorescence assays was blind to the previous *in vivo* history of the animals from which synaptoneurosomes were prepared.

### 2.7 Selective pharmacological agents

**FIGURE 1:**
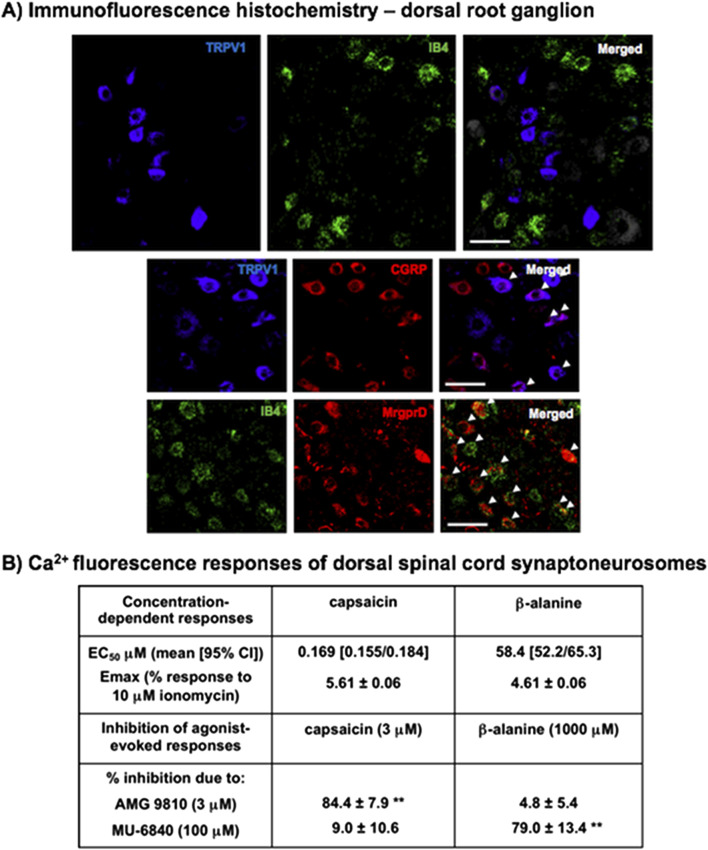
Characterisation of capsaicin and β-alanine as activators of distinct populations of nociceptive afferents. **(A)** Distinct localisation of TRPV1 and MrgprD on different subsets of nociceptive afferents. Typical immunofluorescence images of dorsal root ganglion afferent cell bodies, illustrate very little overlap between TRPV1 and IB4 staining, as well as strong colocalization between TRPV1 and the peptidergic marker, CGRP, or between IB4 and MrgprD (examples indicated by arrowheads). Scale bars represent 50 μm. Quantification of DRG cell staining was carried out on images from 4 separate experiments, with cell counts from 71–196 in each case. **(B)** Ca^2+^ fluorescence responses of naïve lumbar dorsal spinal cord (DSC) synaptoneurosomes to selective TRPV1 and MrgprD activators, capsaicin and β-alanine, applied *in vitro*. Both capsaicin and β-alanine elicited concentration-dependent increases in Ca^2+^ fluorescence, to a similar maximal extent, that were almost completely prevented by the selective TRPV1 antagonist, AMG 9810 (3 μM) and the selective MrgprD antagonist, MU-6840 (100 μM) respectively, without discernible effects on opposing stimuli, **p < 0.01 by One-Way ANOVA with Dunnett’s test in each case. Values are means ± SEM from 6 separate experiments.

Pharmacological agents with the optimum available receptor selectivity were obtained from Tocris, MedChemExpress, Axon MedChem, Key Organics, BOC Sciences or MolPort. In order to activate presynaptic terminals expressing TRPV1 (presumed peptidergic small nociceptive afferents) in DSC synaptoneurosomes, we used the selective TRPV1 agonist, *E*-capsaicin (Caterina et al., 1997). For activation of MrgprD-expressing (presumed non-peptidergic small nociceptor afferents), we used the selective excitatory agonist, β-alanine (Crozier et al., 2007; Rau et al., 2009; Shinohara et al., 2004). Selective antagonists for TRPV1, AMG 9810 (Gavva et al., 2005) and for MrgprD, MU 6840 (Uno et al., 2012) were used to confirm the specificity of targeting by the activators (Figure 1). AMPA-type glutamate receptors were activated by *RS*-AMPA in the presence of cyclothiazide to minimise desensitisation (Kessler et al., 2000) and for the broad spectrum activation of small nociceptive afferents we used the selective GluK1 agonist, 5-iodowillardiine (Jane et al., 1997) in the presence of concanavalin A to minimise desensitisation (Mitchell et al., 2019). (+)-MK 801 was used as a highly selective non-competitive NMDA-type glutamate receptor blocker to confirm induced spinal hypersensitivity (Latremoliere and Woolf, 2009). The potent and selective TRPM8 agonist, icilin, which shows more than 1000-fold selectivity over TRPA1 (Behrendt et al., 2004; McKemy et al., 2002; Story et al., 2003) was used by topical application to the skin to activate TRPM8-positive afferents and elicit spinal inhibitory modulation of nociceptive inputs (Proudfoot et al., 2006). Antagonists (and PAMs where indicated) selective for particular Group II/III mGluRs were used at concentrations as cited in the literature, consistent with affinities specified on the IUPHAR database and shown in pilot experiments to effectively reverse (or potentiate) effects of corresponding agonists on AMPA-induced responses of DSC synaptoneurosomes.

### 2.8 Statistics

Statistical analysis was carried out using GraphPad Prism. All data sets passed the Kolmogorov-Smirnov (KS) normality test. Non-linear curve-fitting (Figure 1B) was carried out using a sigmoidal dose-response (variable slope) model. One-Way ANOVA data sets passed Bartlett’s test for equal variances and were analysed using Tukey’s or Dunnett’s *post hoc* tests, accounting for multiple comparisons. All sample sizes reported in Figure legends represent independent animals unless otherwise specified (total cell counts in Figure 1A).

## 3 Results

### 3.1 Targeting TRPV1 with capsaicin and MrgprD with β-alanine to interrogate distinct subsets of nociceptor input to dorsal spinal cord

Immunofluorescence imaging of nociceptive afferent cell bodies in dorsal root ganglion (Figure 1A) showed that TRPV1 staining is largely distinct from the marker for non-peptidergic nociceptors, IB4 (only 16.2% ± 2.3% of TRPV1^+^ cells showed co-localisation with IB4). In addition, it shows strong overlap between TRPV1 and the nociceptor peptide, CGRP (80.7% ± 4.8% of TRPV1^+^ cells showed co-localisation with CGRP), as well as consistent matching between IB4 and MrgprD (79.8% ± 1.1% of IB4^+^ cells showed co-localisation with MrgprD). This confirms that the majority of TRPV1^+^ and MrgprD^+^ afferents are distinct and therefore that our capsaicin and β-alanine stimuli are likely to be interrogating different subsets of nociceptive afferents.

Measuring Ca^2+^ fluorescence responses of synaptoneurosomes from lumbar DSC, capsaicin and β-alanine elicited robust concentration-dependent increases with EC_50_ values (mean [95%CI]) of 169 [155/184] nM and 58.4 [52.2/65.3] μM and maximal effects of 5.61% ± 0.06% and 4.61% ± 0.06% of those to the internal positive control, 10 μM ionomycin (Figure 1B). These maximal responses each represented around 30%–40% of the maximal response to 5-iodowillardiine (10 μM), a selective agonist for GluK1 receptors, which are considered to be widely expressed in the majority of small nociceptive afferents (Mitchell et al., 2019); consistent with the idea that they may reflect two major subsets of these nociceptors. Responses to capsaicin were strongly inhibited by the selective TRPV1 inhibitor, AMG 9810 and responses to β-alanine were prevented by the selective MrgprD antagonist, MU-6840 without any discernible effect on the opposing stimuli, consistent with the anticipated targeting specificity.

Furthermore, responses to capsaicin and β-alanine were 6-8 fold greater in DSC, rather than ventral spinal cord (VSC) synaptoneurosomes (consistent with the pattern of termination of peptidergic and non-peptidergic nociceptor afferents in dorsal horn) and were abrogated by metabolic or structural disruption of the preparations by preincubation with 3 μM bongkrekic acid (an inhibitor of mitochondrial ADP/ATP translocase and electron transport chain function) or 22 μM digitoxin (a detergent disruptor of membranes). In addition, DSC responses to capsaicin and β-alanine were 93.0% ± 10.1% and 95.5% ± 10.5% inhibited respectively, by preincubation with a low concentration (20 nM) of the exocytosis blocker, tetanus toxin (consistent with their likely presynaptic site of action). In contrast, DSC responses to the direct depolarising agent, veratridine (10 μM) and GluA receptor activation with 20 μM AMPA/10 μM cyclothiazide (which are likely to be largely postsynaptic) were minimally affected (9.4% ± 3.2% and 17.5% ± 2.2% inhibition respectively, n = 4-6 in each case).

### 3.2 Characteristics of enhanced responses to capsaicin and β-alanine in dorsal spinal cord synaptoneurosomes following CCI nerve injury

**FIGURE 2:**
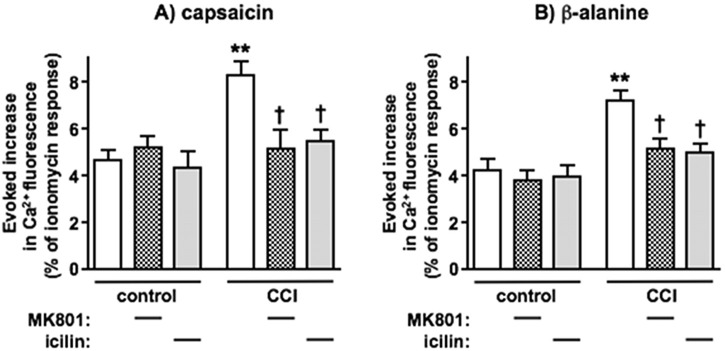
DSC synaptoneurosome responses to capsaicin and β-alanine are both amplified following CCI nerve injury and this increment is reversed by NMDAR antagonist, MK 801 or TRPM8 agonist, icilin. Responses to **(A)** capsaicin and **(B)** β-alanine alone are shown in white, those in the presence of (+)-MK 801 (1 μM, *in vitro*) as hatched and those in the presence of icilin (200 μM, applied topically to the ipsilateral hindpaw) in grey. Statistically significant CCI-induced increments are shown as **p < 0.01 and statistically significant reversals are shown as †p < 0.05 by One-Way ANOVA with Tukey’s test in each case. Values are means ± SEM from 6 separate experiments.

Figure 2 shows that DSC synaptoneurosome Ca^2+^ fluorescence responses to capsaicin (3 μM) and β-alanine (1000 μM) were significantly enhanced (almost 2-fold greater in each case) following CCI nerve injury. The CCI-induced increments were clearly and significantly inhibited by the non-competitive NMDAR inhibitor (+)-MK 801 (1 μM, *in vitro*), consistent with known mechanisms of central sensitisation following nerve injury (Latremoliere and Woolf, 2009) and by topical application to the ipsilateral hindpaw of the TRPM8 agonist, icilin (200 μM); showing that this preparation could reflect the inhibition of pain behaviours seen *in vivo* (Proudfoot et al., 2006). Neither (+)-MK 801 nor icilin had any discernible effect on responses from naïve animal preparations. Although icilin could potentially interact with TRPA1 at high concentrations, its selectivity for TRPM8 is very considerable. As the drug is applied topically here to the surface of unbroken skin, the actual concentration reaching TRPM8^+^ nerve terminals, generally around the epidermal/dermal border (Takashima et al., 2007), will be vastly lower than in the solution being applied. Further, the antinociceptive effect of topical icilin at concentrations as used here is reversed by TRPM8 antisense (Proudfoot et al., 2006) or a highly selective TRPM8 antagonist (Vinuela-Fernandez et al., 2014) and is not mimicked by selective TRPA1 agonists, which actually exert an opposite, pro-nociceptive influence (Proudfoot et al., 2006).

### 3.3 Assessment of TRPM8 agonist reversal of CCI-induced increments in capsaicin- and β-alanine-evoked Ca^2+^ fluorescence responses of dorsal spinal cord synaptoneurosomes – the role of different group II/III mGluRs

**FIGURE 3:**
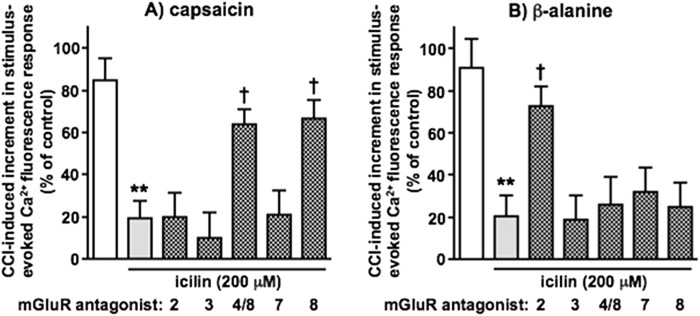
The effects of subtype-selective Group II/III mGluR antagonists on inhibition by icilin of CCI-induced increments in DSC synaptoneurosome responses to capsaicin and β-alanine - selective reversal by mGlu_8_ (and mGlu_4/8_) or mGlu_2_ blockers respectively. CCI-induced incremental responses to **(A)** capsaicin and **(B)** β-alanine alone are shown in white, those in the presence of icilin (200 μM, applied topically to the ipsilateral hindpaw) in grey and those in the presence of icilin plus mGluR antagonists (applied directly to the dorsal surface of lumbar spinal cord) in grey with hatching. The mGlu antagonists used here were Ro 64–5229 (3 μM, mGlu_2_), ML 337 (10 μM, mGlu_3_), MAP4 (250 μM, mGlu_4_/mGlu_8_), VU 6012962 (5 μM, mGlu_7_) and CPPG (3.5 μM, mGlu_8_). Statistically significant inhibition due to icilin, compared to CCI alone, is shown as **p < 0.01 and statistically significant reversals by mGluR antagonists as †p < 0.05 by One-Way ANOVA with Tukey’s test in each case. Values are means ± SEM from 6 separate experiments.

The CCI-induced facilitation of ipsilateral DSC synaptoneurosome responses to both capsaicin and β-alanine was robustly inhibited by topical administration of the TRPM8 agonist, icilin (200 μM) to the paw of the injured hindlimb in anaesthetised animals. mGluR antagonists (in saline with 0.1% DMSO) were applied directly to the dorsal surface of lumbar spinal cord (following preparation of a laminectomy pool at L4-L6) throughout icilin administration (Figure 3). An mGlu_3_ antagonist, ML 337 (Wenthur et al., 2013) and an mGlu_7_ antagonist, VU 6012962 (Reed et al., 2019) had no effect on icilin inhibition of either capsaicin or β-alanine responses. While icilin inhibition of capsaicin responses was similarly unaffected by an mGlu_2_ antagonist, Ro 64–5229 (Kolczewski et al., 1999), the joint mGlu_4_/mGlu_8_ antagonist, MAP4 (De Colle et al., 2000; Han and Hampson, 1999; Knopfel et al., 1995; Pin et al., 1999; Wright et al., 2000) and the selective mGlu_8_ antagonist, CPPG (Hampson et al., 1999; Han and Hampson, 1999; Peltekova et al., 2000) both caused significant attenuation. In contrast, icilin inhibition of β-alanine responses was significantly attenuated by the mGlu_2_ antagonist, but unaffected by the mGlu_4_/mGlu_8_ antagonist or the selective mGlu_8_ antagonist. Similar results were found with further selective mGlu_2_ and mGlu_8_ antagonists, VU 6001966, 1 μM (Bollinger et al., 2017) and MDCPG, 30 μM (Mercier et al., 2013) respectively (data not shown). None of the antagonists had any discernible effect alone on responses in the absence of icilin inhibition. For example, post-CCI capsaicin responses in the presence of Ro 64–5229 and CPPG were 98.3% ± 6.1% and 94.9% ± 5.8% of control respectively, while corresponding β-alanine responses were 101.5% ± 5.0% and 97.6% ± 7.1% of control respectively (values are means ± SEM from 6 separate experiments).

**FIGURE 4:**
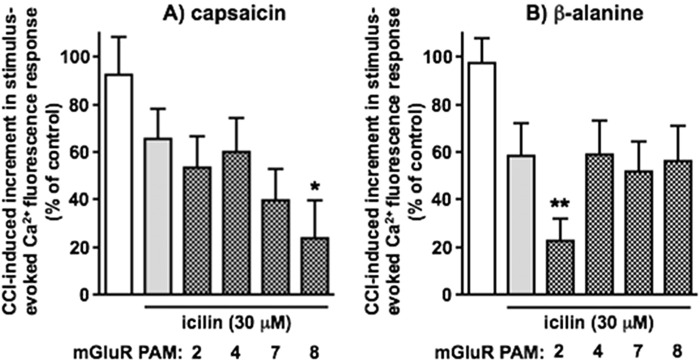
The effects of subtype-selective Group II/III mGluR PAMs on inhibition by icilin of CCI-induced increments in DSC synaptoneurosome responses to capsaicin and β-alanine - selective enhancement by mGlu_8_ or mGlu_2_ PAMs respectively. CCI-induced incremental responses to **(A)** capsaicin and **(B)** β-alanine alone are shown in white, those in the presence of a submaximal concentration of icilin (30 μM, applied topically to the ipsilateral hindpaw) in grey and those in the presence of icilin plus mGluR PAMs (applied directly to the dorsal surface of lumbar spinal cord) in grey with hatching. The mGlu PAMs used here were BINA (1 μM, mGlu_2_), VU 0418506 (30 μM, mGlu_4_), VU 6005649 (10 μM, mGlu_7_) and AZ 12216052 (30 μM, mGlu_8_). Statistically significant inhibition due to icilin in the presence of PAMs, compared to CCI alone, is shown as *p < 0.05 or as **p < 0.01 by One-Way ANOVA with Tukey’s test in each case. Values are means ± SEM from 6 separate experiments.

When a submaximal concentration (30 μM) of icilin was topically administered, capsaicin and β-alanine responses of synaptoneurosomes from CCI animals were not significantly altered. The impact thereon of selective mGluR PAMs (applied directly to dorsal lumbar spinal cord) was then assessed (Figure 4). The inhibitory effect of 30 μM topical icilin only reached statistical significance in the cases of additional mGlu_2_ PAM, BINA (Bonnefous et al., 2005), on β-alanine responses and the mGlu_8_ PAM, AZ 12216052 (Duvoisin et al., 2010; Reed et al., 2013), on capsaicin responses. The selective mGlu_4_ PAM, VU 0418506 (Engers et al., 2016) and the moderately selective mGlu_7_ PAM, VU 6005649 (Abe et al., 2017) did not impact icilin inhibition of either capsaicin or β-alanine responses, while the mGlu_2_ PAM did not affect capsaicin responses and the mGlu_8_ PAM did not affect β-alanine responses. None of the PAMs had any discernible effect alone on responses in the absence of icilin inhibition. For example, post-CCI capsaicin responses in the presence of BINA and AZ 12216052 were 94.3% ± 7.0% and 94.6% ± 7.5% of control respectively, while corresponding β-alanine responses were 95.1% ± 8.2% and 98.9% ± 10.1% of control respectively (values are means ± SEM from 6 separate experiments). mGlu_3_ was not investigated as no highly selective mGluR_3_ PAM, without additional effects on mGluR_2_, is available and the lack of effect of mGluR_3_ antagonist in Figure 3 pointed to any major role in TRPM8 effects being unlikely.

Taken together, the results from Figures 3, 4 indicate a selective role for mGlu_8_ in TRPM8 agonist-induced inhibition of capsaicin-sensitive nociceptive inputs and a selective role for mGlu_2_ in TRPM8 agonist-induced inhibition of β-alanine-sensitive nociceptive inputs.

### 3.4 *In vivo* behavioural reflex assessment of different group II/III mGluR subtypes to TRPM8 reversal of nociceptive hypersensitivity following nerve injury

**FIGURE 5:**
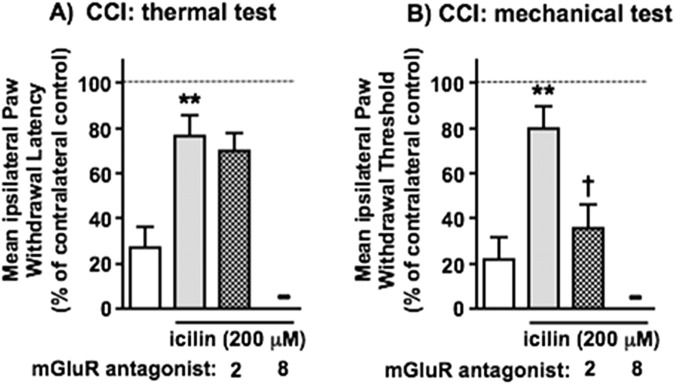
The effects of mGluR antagonist on the inhibition by icilin of CCI-induced hypersensitivity in paw-withdrawal behaviours–selective reversal of reflex mechanical changes by mGlu_2_ antagonist. Ipsilateral hypersensitive changes in reflex paw withdrawal to threshold **(A)** thermal and **(B)** mechanical stimuli are shown in white, those in the presence of icilin (200 μM, applied topically to the ipsilateral hindpaw) in grey and those in the presence of icilin plus mGluR antagonist (delivered i. p.) in grey with hatching. The mGlu antagonist used here was Ro 64–5229 (3 mg/kg). Although our previous results had highlighted both mGluR_2_ and mGluR_8_ as of particular interest, no mGluR_8_ antagonist with suitable CNS-access properties was available to be tested here upon systemic administration. Statistically significant reversal of hypersensitivity by icilin is shown as **p < 0.01 and statistically significant attenuation of this as †p < 0.05 by One-Way ANOVA with Tukey’s test in each case. Values are means ± SEM from 5 separate experiments.

**FIGURE 6:**
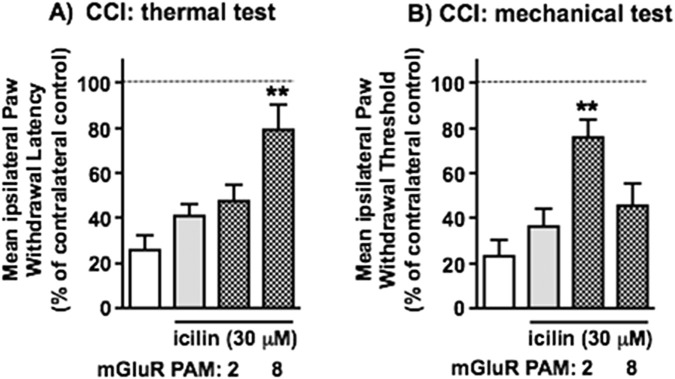
The effects of mGluR PAMs on the inhibition by icilin of CCI-induced hypersensitivity in paw-withdrawal behaviours–selective enhancement of reflex thermal changes by mGlu_8_ PAM and reflex mechanical changes by mGlu_2_ PAM. Ipsilateral hypersensitive changes in reflex paw withdrawal to threshold **(A)** thermal and **(B)** mechanical stimuli are shown in white, those in the presence of a submaximal concentration of icilin (30 μM, applied topically to the ipsilateral hindpaw) in grey and those in the presence of icilin plus mGluR PAMs (delivered i. p.) in grey with hatching. The mGlu_2_ PAM used here was BINA (32 mg/kg) and the mGlu_8_ PAM was AZ 12216052 (10 mg/kg). Statistically significant reversal of hypersensitivity by icilin in the presence of PAMs is shown as **p < 0.01 by One-Way ANOVA with Tukey’s test in each case. Values are means ± SEM from 5 separate experiments.

Figures 5, 6 illustrate the effects of TRPM8^+^ afferent activation on thermal and mechanical nociceptive hypersensitivity behaviours in animals following CCI and the role of key Group II/III mGluRs therein. Figure 5 shows that the reversal of CCI-induced hypersensitivity by topical icilin (200 μM) was significantly attenuated by systemic mGlu_2_ antagonist, Ro 64–5229, in mechanical but not thermal testing. We did not attempt to investigate the role of mGlu_8_ as both of the available selective antagonists (CPPG and MDCPG) are highly polar (very low ACD/LogD, pH 7.4 values) and would be unlikely to reach significant CNS concentrations after i. p. administration. The effects of mGlu_2_ and mGlu_8_ PAMs on the effects of submaximal topical icilin (30 μM) are shown in Figure 6. While this concentration of icilin alone did not significantly attenuate CCI-induced hypersensitivity, the mGlu_2_ PAM, BINA, facilitated the TRPM8 effect on mechanical responses, to reach statistical significance, but did not affect thermal responses. In contrast, the mGlu_8_ PAM, AZ 12216052, amplified the TRPM8 effect on thermal, but not mechanical responses. These findings, at the level of *in vivo* nociceptive behaviours, show a strikingly selective differential role for mGlu_8_ in TRPM8 afferent-evoked attenuation of thermal hypersensitivity, but mGlu_2_ in its attenuation of mechanical hypersensitivity. This aligns closely with observations from capsaicin- and β-alanine-evoked responses of DSC synaptoneurosomes in Figures 3, 4.

### 3.5 Group II/III mGluR-dependent attenuation of nociceptive inputs by TRPM8 at the spinal level impacts processing at higher levels of the CNS

**FIGURE 7:**
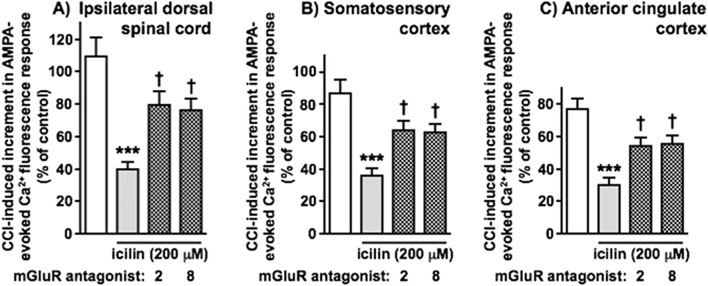
The inhibitory effects of hindpaw topical icilin on CCI-induced increments in synaptoneurosome responses to AMPA at spinal cord and higher levels of the CNS involved in pain processing are attenuated by spinal mGlu_2_ or mGlu_8_ antagonists. Responses to AMPA for **(A)** ipsilateral DSC, **(B)** somatosensory cortex and **(C)** anterior cingulate cortex are shown in white, those in the presence of icilin (200 μM, applied topically to the ipsilateral hindpaw) in grey and those in the additional presence of mGluR_2_ or mGluR_8_ antagonists (applied directly to the dorsal surface of lumbar spinal cord) in grey with hatching. The mGlu antagonists used here were Ro 64–5229 (3 μM, mGlu_2_) and CPPG (3.5 μM, mGlu_8_). Statistically significant inhibitory effects of icilin are shown as ***p < 0.001 and statistically significant reversals by mGluR antagonists as †p < 0.05 by One-Way ANOVA with Tukey’s test in each case. Values are means ± SEM from 6–8 separate experiments.

Figure 7 illustrates CCI-induced increases in responses of synaptoneurosomes from ipsilateral DSC, somatosensory cortex and anterior cingulate cortex. In each case, the CCI-induced increment in responses to AMPA (in the presence of cyclothiazide) was inhibited by topical administration of icilin (200 μM) to the paw of the injured hindlimb and partial (but statistically significant) reversal was seen with either mGlu_2_ antagonist (Ro 64–5229) or mGlu_8_ antagonist (CPPG) applied directly to the dorsal surface of lumbar spinal cord. These findings indicate that the distinct involvement of mGlu_2_ and mGlu_8_ in TRPM8 inhibition of nerve injury-induced hypersensitivity is maintained in terms of reduced excitability at supraspinal levels of pain processing pathways. We did not attempt to investigate PAMs in this experimental setting as the inhibitory effects of even a maximal concentration of icilin were more modest at supraspinal levels.

## 4 Discussion

Our primary aim here was to further elucidate the processes underpinning the analgesic effect resulting from activation of TRPM8^+^ afferents. TRPM8^+^ afferents terminate in superficial dorsal horn, showing partial overlap with CGRP^+^ or Substance P^+^ fibres (but little evidence for direct contact) and almost no overlap with IB4^+^ afferents (Dhaka et al., 2008; Knowlton et al., 2013). A subpopulation of rostrally projecting neurons in lamina I is responsive to innocuous cooling (Christensen and Perl, 1970; Dostrovsky and Craig, 1996; Hachisuka et al., 2020) and may receive direct inputs from TRPM8^+^ afferents (Bell et al., 2024; Lee et al., 2025), although local interneurons also contribute to mediating or modulating the cool sensory pathway (Albisetti et al., 2023; Lee et al., 2025). In contrast to the ascending pathways for cool sensation, the processes underlying the spinal antinociceptive influences of TRPM8^+^ afferents remain little understood. We addressed that here with a pharmacological strategy attempting to differentially interrogate synaptic processes emanating from peptidergic and non-peptidergic nociceptors and investigate the influence of activating TRPM8^+^ afferents on each, together with the underlying mechanisms. The insights gained could potentially point the way to novel therapeutic opportunities for safe and effective analgesia to relieve refractory chronic pain states.

We previously identified that inhibitory Group II or Group III mGluRs in dorsal spinal cord contributed importantly to the inhibitory gating of nociceptive inputs following peripheral or intrathecal administration of TRPM8 agonists (Proudfoot et al., 2006). The advent of highly selective antagonists and PAMs for each of these mGluRs has made it possible to define more closely which mGluRs are responsible and consequently develop further understanding of what could represent more refined analgesic interventions for hypersensitive pain states.

The current study demonstrates initially that TRPV1^+^ and IB4^+^ DRG cell bodies are largely distinct and show strong overlap with CGRP and MrgprD staining respectively (thought to separately label peptidergic and non-peptidergic classes of afferent nociceptors in each case). Synaptoneurosome preparations from DSC showed robust and concentration-dependent Ca^2+^ fluorescence responses to the TRPV1 agonist, capsaicin, and to the MrgprD agonist, β-alanine, providing a tool to interrogate the regulation of different classes of nociceptors by activation of TRPM8^+^ afferents. A nerve injury model (CCI) resulted in incremental responses to capsaicin and β-alanine that could be reversed by either an NMDA receptor antagonist (consistent with established processes of central sensitisation in ongoing pain states) or by topical administration of the TRPM8 agonist, icilin. Icilin reversal of the CCI-induced increment in responses to capsaicin was selectively blocked by mGlu_8_ antagonists, whereas the impact of icilin on responses to β-alanine was selectively blocked by mGlu_2_ antagonists. Corresponding results were found with mGlu_8_ and mGlu_2_ PAMs in facilitating submaximal effects of icilin in each case. *In vivo* reflex paw withdrawal behaviours to thermal and mechanical nociceptive stimuli are thought to be driven by these peptidergic and non-peptidergic nociceptors respectively, and correspondingly the impact of icilin in reversing CCI-induced hypersensitivity was modified by mGlu_8_/mGlu_2_ antagonist/PAMs, in line with the *in vitro* results. TRPM8 reversal of nerve injury-induced nociceptive hypersensitivity was shown to rely on mGlu_8_ activation for thermal sensing and mGlu_2_ for mechanical sensation, a clear indication of distinct parallel processing of modality-specific nociceptive information and that this can potentially be targeted in a differential manner by potential therapeutic interventions. Further experiments indicated that the impact of icilin reversal of CCI-induced hypersensitivity at DSC level (and its modality-specific reliance on mGlu_8_ or mGlu_2_) is replicated through pain processing regions at higher levels of the CNS; somatosensory and anterior cingulate cortex. One technical limitation is that these experiments were carried out solely in male rats, in order to best relate to findings in our previous work. We therefore cannot say whether mechanistic differences in selective mGluR gating of particular nociceptive afferent inputs can be extrapolated to females, although we do know that TRPM8 analgesia in human neuropathic pain patients is clearly apparent in both genders (Fallon et al., 2015). Extensive further study would be required to address any potential sexual dimorphism in TRPM8 analgesia here and the role of mGluRs therein, potentially even considering oestrous cycle stage and the role of steroids as further variable factors.

Group II and Group III mGluRs (with the exception of mGlu_6_) are widely expressed throughout the central nervous system, where they exert primarily inhibitory influences and may be located pre- or postsynaptically (Gregory and Goudet, 2021). Immunohistochemical studies report the presence of mGlu_2/3_ in superficial dorsal horn, including on afferent terminals, as well as in small DRG neurons, especially those binding IB4; i.e., presumed non-peptidergic nociceptors (Carlton et al., 2001; Jia et al., 1999; Petralia et al., 1996; Tang and Sim, 1999). mRNA for mGluR_3_ (associated with glial and neuronal profiles), is expressed in DSC, while mGlu_2_ mRNA was not detected (Okubo et al., 2019). Both mRNA and immunoreactivity for mGluR_4_ and mGluR_7_ are present in DRG and superficial dorsal horn (Azkue et al., 2001; Li et al., 1997; Ohishi et al., 1995; Okubo et al., 2019). Immunoreactivity for mGlu_8_ has been identified particularly in small DRG neurons that are TRPV1-positive and thus may be predominantly peptidergic C-fibre nociceptors (Govea et al., 2012).

Our studies do not provide direct insights into the precise location of the mGlu_2_ and mGlu_8_ receptors here, other than to indicate selective impact on first relay synapses from non-peptidergic and peptidergic afferents. These mGluRs could in principle be located presynaptically, postsynaptically, perisynaptically, or indeed extrasynaptically (Pal, 2018); mGlu_2,_ for example, showing a preferred extrasynaptic localisation compared to a defined perisynaptic clustering for postsynaptic mGlu_1_/mGlu_5_ (Azkue et al., 2000; Lujan et al., 1997), as well as notable presynaptic mobility to sites outside the active zone, in contrast to mGlu_7_ (Bodzeta et al., 2022). The glutamate released here (directly or indirectly) due to activation of TRPM8^+^ afferents may not necessarily act through direct synaptic contacts, but instead by synaptic spillover (“volume transmission”) to reach mGluRs at more distant perisynaptic/extrasynaptic sites (Hires et al., 2008; Isaacson, 1999; Pal, 2018; Rusakov and Stewart, 2021; Scanziani, 2002; Vogt and Nicoll, 1999). The reports of mGlu_2/3_ immunoreactivity associated with IB4^+^ afferents and mGlu_8_ immunoreactivity associated with TRPV1^+^ afferents could be consistent with their extrasynaptic localisation on presynaptic profiles of the respective subtypes of nociceptive afferent synaptoneurosomes here, although there clearly may be alternative explanations for our observations. Further immunohistochemical studies could be carried out to investigate the distribution of mGluR_2_ and mGluR_8_ in relation to TRPV1^+^ and MrgprD^+^ terminals, although precise synaptic localisation would be difficult to ascertain other than perhaps at EM level, and to date, we have not been able to access antibodies with the specificity needed. In terms of any potential mechanistic differences, mGlu_2_ and mGlu_8_ are thought to show similar affinity for their endogenous ligand, L-glutamate (Cartmell et al., 1998; Peltekova et al., 2000; Schweitzer et al., 2000), and similar G protein signalling through Gi/Go leading to inhibition of adenylyl cyclase and likely regulation of potassium and calcium channels (Flor et al., 1995; Wu et al., 1998). However, they appear to show marked differences in desensitization and interaction with G protein-coupled receptor kinases (GRKs) and arrestins; mGlu_2_ being notably resistant to agonist-induced desensitization, while mGlu_8_ readily engages in GRK/arrestin interactions, leading to desensitization and potentially alternative signalling (Abreu et al., 2021; Iacovelli et al., 2009; Marx et al., 2025; Zhao et al., 2025).

Apart from their specific spinal cord role in TRPM8 analgesia addressed here, Group II/III mGluRs are widely reported to exert antinociceptive effects more broadly (Chiechio, 2016; Pan et al., 2008), acting at peripheral (Carlton et al., 2011; Du et al., 2008; Govea et al., 2012; Lee et al., 2013; Park et al., 2019), DRG (Davidson et al., 2016; Sheahan et al., 2018) or spinal cord (Chen and Pan, 2005; Fisher et al., 2002; Gerber et al., 2000; Goudet et al., 2008; Neugebauer et al., 2000; Zhang et al., 2009; Zhou et al., 2011) sites. With regard to Group II mGluRs, mGlu_2_^−/−^, but not mGlu_3_^−/−^ mutant mice show increased responses to nociceptive stimuli and diminished antinociceptive effects of a dual mGlu_2/3_ agonist (Fujioka et al., 2014; Zammataro et al., 2011). Studies targeting specific Group III mGluRs point to spinal antinociceptive roles of mGluR_4_ (Goudet et al., 2012; Vilar et al., 2013; Wang et al., 2011), mGluR_7_ (Dolan et al., 2009; Mitsukawa et al., 2005; Osikowicz et al., 2008; Wang et al., 2021) and mGluR_8_ (Gerber et al., 2000; Govea et al., 2012; Okubo et al., 2019). Clearly, a number of Group II/III mGluRs can potentially exert spinal antinociceptive influences, but the current findings explicitly identify mGluR_2_ and mGluR_8_ in particular as mediators of TRPM8-elicited attenuation of spinal nociceptive transmission, with differential impact on distinct parallel nociceptive inputs. While the present study is focused on a strategy to impact pain processing at the spinal cord level, pre-empting nociceptive transmission to higher levels of the CNS, it is possible that additional supraspinal effects of systematically administered mGluR_2_/mGluR_8_ agents (Chiechio, 2016; Pan et al., 2008) could interact with spinal cord mechanisms and modify potential therapeutic outcomes.

**FIGURE 8:**
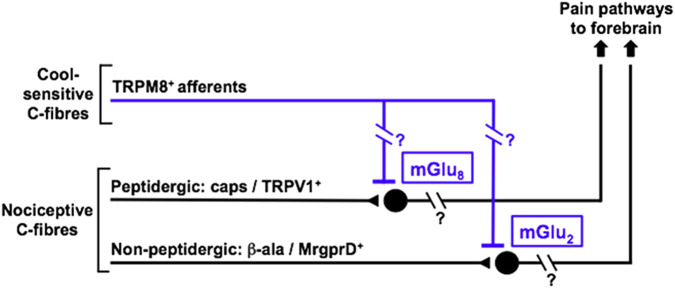
Schematic diagram outlining the differential roles of mGlu_2_ and mGlu_8_ in TRPM8^+^ afferent-induced inhibition of transmission from distinct classes of nociceptor inputs to spinal cord.

Overall, this study provides further evidence that neuropathic hypersensitivity in spinal nociceptive processing can be suppressed by concurrent activation of TRPM8^+^ afferents, together with showing for the first time that thermal and mechanical subsets of nociceptors are differentially regulated in this process by distinct mGluR subtypes (Figure 8). This strongly supports the fundamental concept of different nociceptive modalities undergoing parallel processing and highlights the opportunity for highly selective intervention according to need, i.e., personalised approaches to achieving optimal pain relief. Despite the use of several linked modes of investigation; immunohistochemistry, *ex vivo* synaptoneurosome functional responses and reflex pain behaviours, there are limitations to the range of information provided. While all neural circuitry is intact to mediate mechanisms of TRPM8-induced antinociception up until the point of synaptoneurosome preparation, the anatomical arrangements are then lost, so assessments rely then on direct impacts on the synaptoneurosomes whose responses are being measured. Secondly, our interrogation of these processes is essentially pharmacological, so relies very much on the selectivity of the available compounds, although we have deployed these in a rigorous manner throughout. Additionally, there could be a small degree of expression of the receptors we have used to interrogate nociceptors at sites in dorsal horn other than on primary afferent nociceptor terminals (Zhou et al., 2009). Further, there is increasing evidence for mGluRs functioning in heterodimeric as well as homodimeric complexes (Doumazane et al., 2011; Lee et al., 2020) and some possible pharmacological distinctions between these (Kammermeier, 2012; Lin et al., 2024; McCullock et al., 2025), but tools are not available for us to define the precise molecular composition of the Group II/III mGluR complexes underlying the current observations.

## 5 Conclusion

In summary, this work provides clear indications that GluR_2_ and mGluR_8_ play distinct roles in TRPM8-mediated analgesia, modulating neuropathic mechanical and thermal hypersensitivity respectively. As well as giving new insights into fundamental biology, this points to the potential for novel subtle interventions, such as mGluR_2_ and mGluR_8_ agonist-evoked analgesia or GluR_2_/mGluR_8_ PAM facilitation of different facets of TRPM8 analgesia. More refined personalised analgesic interventions could become possible along these lines, reinforcing a general principle by which the full impact of not only TRPM8, but also other potential non-opioid analgesic targets may be more effectively realised.

## Data Availability

The raw data supporting the conclusions of this article will be made available by the authors, without undue reservation.
